# Why be red listed? Threatened Myriapoda species in Brazil with implications for their conservation

**DOI:** 10.3897/zookeys.741.21971

**Published:** 2018-03-07

**Authors:** Manoela Karam-Gemael, Thiago Junqueira Izzo, Amazonas Chagas-Jr

**Affiliations:** 1 Programa de Pós Graduação em Ecologia e Conservação da Biodiversidade, Universidade Federal de Mato Grosso, Avenida Fernando Corrêa da Costa, s/n, CEP: 78060-900, Cuiabá, Mato Grosso, Brasil; 2 Departamento de Botânica e Ecologia, Universidade Federal de Mato Grosso, Avenida Fernando Corrêa da Costa, s/n, CEP: 78060-900, Cuiabá, Mato Grosso, Brasil; 3 Departamento de Biologia e Zoologia, Universidade Federal de Mato Grosso, Avenida Fernando Corrêa da Costa, s/n, CEP: 78060-900, Cuiabá, Mato Grosso, Brasil

**Keywords:** caves, centipedes, conservation management, ecotourism, extinction risk, IUCN, millipedes, protected areas, public policies, tropical region

## Abstract

The biodiversity crisis we live in, marked by high extinction rates, requires well-planned conservation efforts. To overcome this issue, red lists of threatened species are recognized as the main objective approach for evaluating the conservation status of species and therefore guiding conservation priorities. This work focuses on the Myriapoda (Chilopoda and Diplopoda) species listed in the Brazilian red list of fauna to enable discussion of the practical implications of red lists for conservation. Almost all myriapods assessed are endemic to Brazil (99 %) and 73 % are known from subterranean habitats only. Despite of 33 % being recorded from protected areas (PAs), downgrading, degazettement or downsizing of PAs and intense and unregulated ecotourism represent great threats. The PAs network in Brazil tends to fail in conserving myriapod species. The number of data deficient species (42 %) states the need of investing in ecological and taxonomic studies about the group, in order to fill in important knowledge gaps in species assessments nationally and globally. In this work we show that there is a lack of communication between national and global agencies concerning red lists, which results in a significant loss for science and for conservation. Despite investing in national and state red lists, individual countries must take the final step of submitting its data to IUCN global database, as significant international funding is available for IUCN red listed species conservation. Being one of the most diverse countries in the world, and facing the biggest cuts ever on national science funding, losing these important funding opportunities is a huge loss for Brazilian biodiversity conservation and for science. This study raises awareness on subterranean habitats conservation, due to its high endemism and fragility. Since the first edition of the Brazilian Red List in 1968, centipedes are now included for the first time, and millipedes for the second time. The presence of these myriapods in the list brings attention to the group, which usually receives little or no attention in conservation programs and environmental impact assessments. Rather than a specific case for Myriapoda and for Brazil, the points discussed here can be related to arthropods and the tropics, as the most biodiverse countries are emerging economies facing similar challenges in PAs network management, species extinction risks and science funding.

## Introduction

Species extinction has always been part of biodiversity history. But recent extinction rates are 100 to 1000 times their pre-human levels in well-known and taxonomically diverse groups from widely different environments (Pimm et al. 1995). The overarching driver of species extinction is human population growth and increasing per capita consumption. How long these trends continue, where and at what rate, will dominate the scenarios of species extinction and challenge efforts to protect biodiversity (Pimm et al. 2014).

To understand and prevent human-driven extinction processes in progress, it is reasonable to know current living species diversity and distribution, in order to evaluate their probability of extinction. The red lists of threatened species are recognized as the most objective approach for evaluating the conservation status of species (IUCN 2013), and they represent the primary source of information to establish a species conservation status following defined protocols (Lewinsohn et al. 2005, Mallon and Jackson 2017). Red lists gather essential scientific evidence required to guide strategic and financial biodiversity conservation planning, the formulation of environmental public policies and conservation priorities and trends. Red lists are also indicators of data gaps in taxonomic groups or regions, orientating new biodiversity research. For example, a high number of species classified as Data Deficient shows that there is not enough knowledge about a given taxonomic group. Although inclusion in a red list is an indication of actual threat, absence of an entire taxonomic group from the list should be treated with circumspection because its omission could result from a lack of information rather than the absence of threat (Lewinsohn et al. 2005).

Given the growing concern about environment conservation, governments and/or environmental NGOs have been working in local conservation initiatives. Individual countries' red lists are constructed in regional or national levels and may inform local to global conservation decisions (Byrne and Fitzpatrick 2009). Red lists are implemented officially throughout environmental public policies at national and state levels across the countries. Usually they are funded by state or national governments, and coordinated by its environmental agencies. In Brazil, the process of the list construction involves an extensive literature review by specialists, followed by workshops to discuss and validate each species assessments details and criteria.

On the other hand, the IUCN (International Union for Conservation of Nature) Red List is considered the international authority for assessing species’ extinction risk, informing global to local conservation decisions (Ocampo-Peñuela 2016). The list construction is based on a protocol that classifies species into different categories of risk using a formal set of objective and standard criteria (IUCN 2017a). The process regularly updates species status, and all the associated data are publicly accessible. Certainly, individual countries make their own decisions and may set management policies based on the IUCN assessments (Ocampo-Peñuela 2016). Both national lists and IUCN global assessments are primary information sources and may be complementary to each other on conservation programs.

### Threatened myriapods in red lists

Despite their relevant ecosystem services and functions, in general arthropods are poorly represented in conservation assessments (Lewinsohn et al. 2005, Diniz-Filho et al. 2010, Cardoso et al. 2011), which hinder an in-depth analysis of their conservation status (Lewinsohn et al. 2005). However, comprehensive biodiversity studies need to include as many taxa as possible (Oliveira et al. 2017). Considering invertebrates' high abundance and diversity worldwide, studies extending its knowledge and helping to fill in its scientific gaps are really necessary to its conservation and, therefore, to ecosystems services conservation in the long run.

The Myriapoda includes four classes: Chilopoda, Diplopoda, Pauropoda, and Symphyla. The myriapod fauna known for Brazil encompasses mainly Chilopoda (134 described species (Chagas-Jr 2017)) and Diplopoda (536 described species (Pena-Barbosa 2017)). It is estimated that there are around 400 Chilopoda species and 5,000 Diplopoda species only in the Amazon Forest (Adis and Harvey 2000). Pauropoda and Symphyla are almost unknown to science, and estimates indicate that there are fewer than 200 species of Pauropoda and fewer than 20 species of Symphyla in the Amazon Forest (Adis and Harvey 2000). Myriapods are widely distributed in Brazil and can be easily found in urban areas. Scolopendromorphs are most responsible for accidents with humans and their venom has been studied due to its medical interest, the novelty of its protein and peptide composition (Undheim et al. 2015) and potential for pharmacology (Harvey 2014; Hakim et al. 2015; Undheim et al. 2016). In China centipedes are one of the crucial venomous arthropods that have been used in traditional medicine for hundreds of years (Hakim et al. 2015).

**Figure 1.**
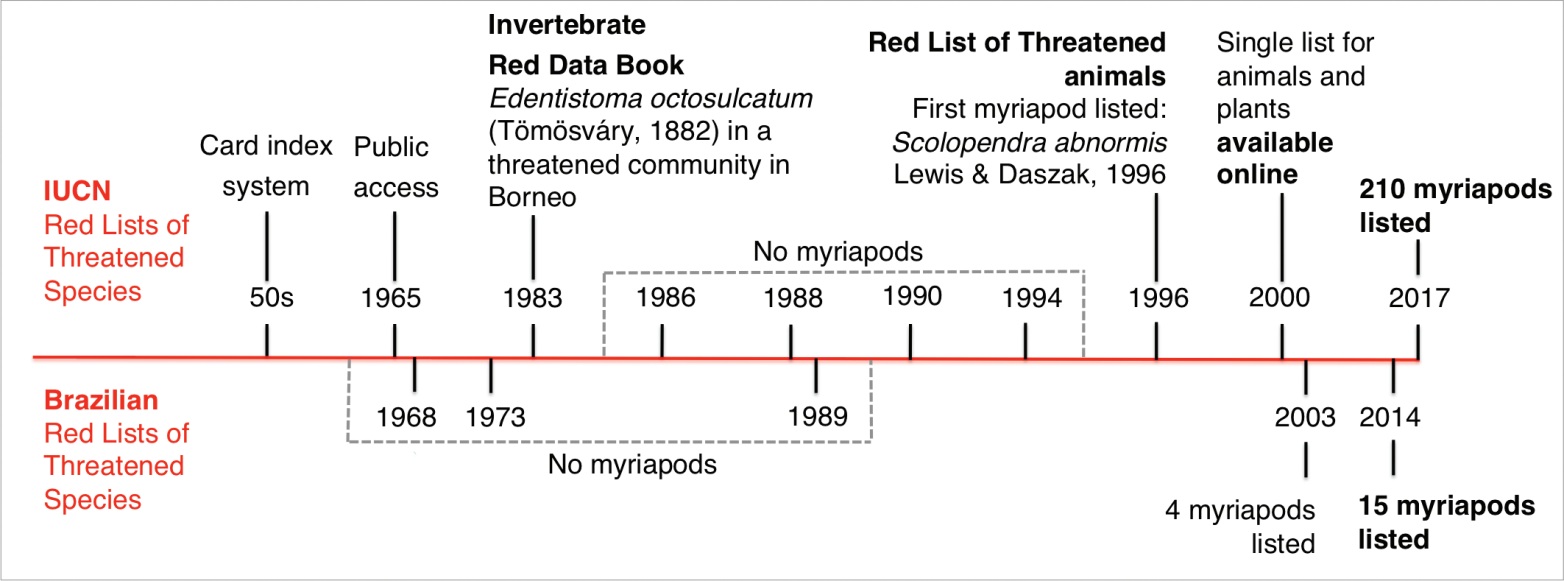
Myriapoda in the IUCN and Brazilian red lists. Timeline of Myriapoda species included in the IUCN Red Lists of Threatened Species (above the red line), and in the Brazilian Red Lists of Threatened Species (below the red line), highlighting the first myriapods listed and the current number of species listed.

Invertebrate animals were not initially included in red lists. The early beginnings for the IUCN Red List started in the 1950s with a card index system documenting data on threatened mammals and birds (Figure 1). In 1965 the first most comprehensive lists of threatened mammals and birds were published – enabling public access to the data for the first time. Since then, IUCN published several versions of its red lists encompassing mammals, birds, amphibians, reptiles, fishes, and several lists focused also on plant species. Invertebrates were first evaluated for the IUCN Red List in 1983, when The IUCN Invertebrate Red Data Book was published. Although this list presents all four classes of Myriapoda (Chilopoda, Diplopoda, Symphyla, and Pauropoda), and mention its scientific interest and threats to survival, the species were not yet assessed individually at that time. The IUCN Invertebrate Red Data Book also assessed some biological communities as a whole, where entire sets of invertebrates were in need of conservation. In Gunung Mulu National Park, in Borneo, the bizarre and rare centipede *Edentistoma octosulcatum* (Tömösváry, 1882) is listed in a threatened community. The first myriapod specifically assessed and listed in the IUCN Red List, according to the historical publications available at the institution website, was *Scolopendra abnormis* Lewis & Daszak, 1996, classified as vulnerable with a very small population. Since 2000 the IUCN Red List is available online (http://www.iucnredlist.org/) and nowadays it includes 200 millipede and ten centipede species.

In Brazil, the first national red list was published in 1968 (Figure 1), but it was only in the 2000 decade that the Brazilian lists adopted international standards of species assessments, using IUCN method, criteria and categories. Invertebrate assessments have been included in Brazilian red lists recently (Figure 1). The first myriapods included in a Brazilian red list were four millipede species in the 2003 list (*Leodesmus yporangae* (Schubart, 1946), *Peridontodesmella alba* Schubart, 1957, *Yporangiella stygius* Schubart, 1946, and *Rhinocricus padbergi* Verhoeff, 1938). The current Brazilian red list was published in 2014 and it includes 15 myriapod species (12 millipedes and three centipedes) (MMA 2014).

Given the continental size and great biodiversity of Brazil, it is unsurprising that sampling coverage is very unequal among biomes and taxonomic groups (Lewinsohn et al. 2005). Both in the IUCN Red List and in the Brazilian lists, invertebrate animal assessments have always been uneven to vertebrate animals. For example, the 2017 IUCN Red List (version 2017-1) evaluated 1 % of invertebrates and 66 % of vertebrates of all described species. Even taking into account that the number of described species explains part of this unevenness (1.3 million for invertebrates and 68,000 for vertebrates (IUCN 2017b)), the number of species evaluated emphasizes invertebrate negligence (19,000 for invertebrates and 45,000 for vertebrates). Similarly, the current Brazilian Red List (2014) evaluated 3 % of invertebrates and 99 % of vertebrates described (3,000 invertebrate and 9,000 vertebrate species). However, this quantitative similarity between invertebrate and vertebrate proportions in Brazilian and IUCN red lists may hide an important qualitative mismatch between the lists, which can be a product of the lack of communication between national and international agencies. A focus on the implications of 2014 Brazilian Red List data for myriapods (Chilopoda and Diplopoda) conservation in Brazil allows a discussion of the current context and the relative effectiveness of the red lists of threatened species for biodiversity conservation in Brazil. Additionally, the implication of the discrepancies between the Brazilian red list and the IUCN list and the effectiveness of protected areas (PAs) Brazilian network in conserving threatened myriapods is discussed.

## Materials and methods

The current Brazilian red list of threatened species of fauna was constructed through specialists workshops held by ICMBio (Chico Mendes Institute of Biodiversity Conservation, a national agency of the Brazilian Ministry of Environment) and it was published as a legal act in December 17, 2014 (MMA 2014). In 2016, ICMBio also published the Executive Summary of the Brazil Red Book of Threatened Species of Fauna, which includes more information about the threatened species listed in 2014 (MMA 2016). The assessments workshops followed IUCN methods, categories and criteria to assess species, which classifies the extinction risk as Critically endangered (CR), Endangered (EN), Vulnerable (VU), Near threatened (NT), Least concern (LC), and Data deficient (DD). The categories CR, EN and VU are considered the threatened ones.

**Figure 2.**
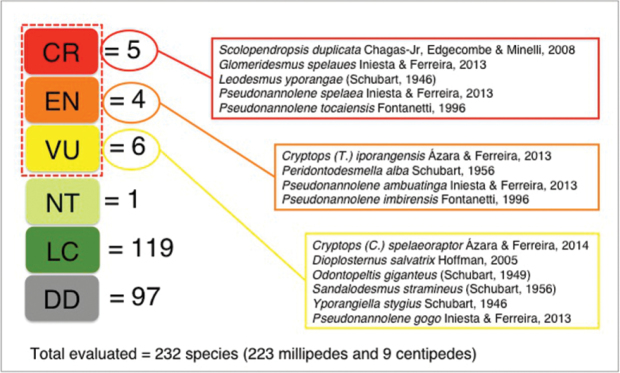
Myriapoda species assessement by the current Brazilian Red List. Myriapoda threatened species according to the 2014 Brazilian Red List, which follows IUCN classification categories (CR = Critically endangered, EN = Endangered, VU = Vulnerable, NT = Near threatened, LC = Least concern, DD = Data deficient). Dashed red line indicates threatened categories.

This study focused on the Myriapoda species in the 2014 Brazilian red list (Figure 2) and its related data available on the Executive Summary published in 2016. The analysis consisted of a qualitative comparison between the species listed in the 2014 Brazilian red list and those listed in the IUCN Red List (version 2017-1, http://www.iucnredlist.org/). The software QGIS (version 2.18.7) was used to create the map using Brazilian biomes and protected areas shape files, besides Myriapoda threatened species distribution data. Both biomes and protected areas shape files were downloaded from the Brazilian Environment Ministry website (http://mapas.mma.gov.br/i3geo/datadownload.htm) in June 2017. Myriapoda threatened species geographic coordinates were compiled from the original descriptions' publications (See Suppl. material 1: Myriapoda threatened species geographic coordinates).

## Results

The Brazilian red list encompasses more Myriapoda families and genera than IUCN red list, especially for Diplopoda species (Table 1). Comparing the families, four Diplopoda families (Paradoxosomatidae, Pyrgodesmidae, Siphonophoridae, Spirostreptidae), and two Chilopoda families (Ballophilidae and Scolopendridae) are shared between the two lists. Concerning the genera, only one of each class is included in both the IUCN and the Brazilian lists: *Rhinocricus* (Diplopoda), and *Ityphilus* (Chilopoda). There are no shared myriapod species between the IUCN and the Brazilian red lists.

**Table 1.** Myriapoda diversity in IUCN Red List (2017) and in Brazil Red List (2014), including all extinction risk categories: Critically endangered, Endangered, Vulnerable, Near threatened, Least concern, and Data deficient.

Reference	Diplopoda			Chilopoda		
	Families	Genera	Species	Families	Genera	Species
**IUCN Red List**	12	35	200	5	5	10
**Brazil Red List**	17	76	223	6	7	9
**Shared taxa**	4	1	0	2	1	0

**Figure 3.**
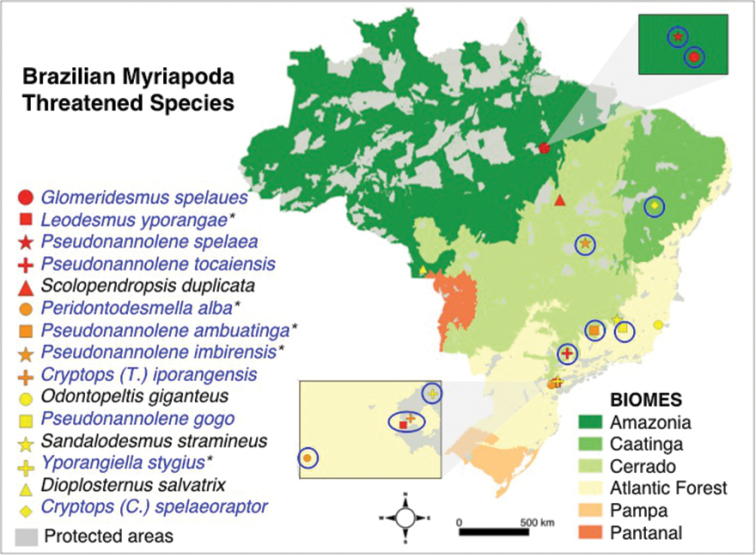
Distribution of the Brazilian Myriapoda threatened species. The color of the legend represents the IUCN threatened category: red (Critically endangered – CR), orange (Endangered – EN), and yellow (Vulnerable – VU). Species in blue are only known from subterranean habitats. Species with an asterisk (*) occur inside PAs.

Almost all myriapods species assessed for the Brazilian red list are endemic to Brazil (99 %), and so are all of those classified as threatened (100 %). Among the species categorized as threatened, 73 % are only known for subterranean habitats (Figure 3), and just 33 % occurs inside PAs. Concerning the Brazilian biomes, 40 % of threatened myriapod species are in Atlantic Forest, 33 % in Cerrado, 20 % in Amazonia, and 7 % in Caatinga (Figure 3).

Concerning the species classified as Data Deficient (DD), 98 % refers to Diplopoda and just 2 % refers to Chilopoda (Table 2). Among Diplopoda, the order Polydesmida encompasses the highest number of DD species in Brazil. Concerning the subterranean myriapod fauna, Spirostreptida is the order more frequently recorded (Table 2).

**Table 2.** Myriapoda orders represented among Data Deficient (DD) species and cave species in the 2014 Brazilian Red List of Threatened Species.

Class	Order	DD species	Cave species
Diplopoda	Polydesmida	51 %	27 %
	Spirobolida	25 %	0 %
	Spirostreptida	21 %	46 %
	Siphonophorida	1 %	0 %
	Glomeridesmida	0 %	9 %
Chilopoda	Scolopendromorpha	2 %	18 %
	Geophilomorpha	0 %	0 %
	Scutigeromorpha	0 %	0 %
	Lithobiomorpha	0 %	0 %

## Discussion

### What is the importance of a species being included in a red list?

There are some implications of a species being included in a red list. First, the assessment data itself have an intrinsic value of knowing biodiversity status in a given period of history and its associated extinction risks (Dijkstra 2017). At each update of the list, new species are assessed, compiled, and summarized and, thus, more knowledge is generated about the group itself. This kind of data also allows temporal assessment of the species populations (Schachat et al. 2015, Cruickshank et al. 2016). Second, when a species is included in a red list it gets attention and becomes among the priorities for conservation efforts, once red lists gather scientific evidence required to guide biodiversity conservation planning, the formulation of public policies and conservation priorities and trends (Mallon and Jackson 2017). Conservation science being an applied discipline, red lists operate like the first step to the management of species. Third, but not less important, the inclusion of a species in a red list increases the possibility of raising funds to study the species (but on the other hand, bureaucratic obstacles also increase).

Science funding in Brazil has been suffering huge cuts at federal and state levels in recent years, which have paralyzed research (Gibney 2015). From electric and cleaning expenses to laboratories working and field research and meetings, science and research institutions do not have enough funds to pay the basics, and face one of the worst science funding crisis to strike Brazil in decades (Escobar 2015). Besides paralyzing research in Brazil, after a decade of economic boom and its investments resulting in high quality science (Gibney 2015), Brazil is also facing the loss of scientists that have opportunity to live and work abroad; Brazilian science is bankrupt (Escobar 2015).

Once conservation efforts are limited and priorities must be set, in practice red lists work as a priority indicator for conservation investments. In Brazil there are calls for biodiversity conservation directed specifically to threatened species, i.e. Fundação O Boticário (http://www.fundacaogrupoboticario.org.br). For those, the presence of a given species in the Brazilian red list is the main criteria for funding eligibility. Similarly, there are international calls directed to fund research and conservation programs of species assessed for the IUCN Red List. There are several small grants provided by scientific associations that potentially fund postgraduate research, i.e. Whitley Fund for Nature (https://whitleyaward.org/), The Rufford Foundation (https://www.rufford.org/rsg/), Saving Species (http://www.savingspecies.org/), People’s Trust for Endangered Species (https://ptes.org/). There are also bigger agencies providing grants to entire conservation programs. For example, SOS – Save Our Species (http://www.saveourspecies.org/) is a joint initiative of the IUCN, the Global Environment Facility, and the World Bank. Its objective is to ensure the long-term survival of threatened species and their habitats, supporting direct action on species conservation priorities informed by the IUCN Red List of Threatened Species, among other criteria. Between 2010 and 2016, the SOS initiative allocated US$ 10 million to species conservation, encompassing 250 threatened species in more than 50 countries (including Brazil in a critically endangered bird project in 2010). Another example is The Mohamed bin Zayed Species Conservation Fund (https://www.speciesconservation.org/), a private institution that invested US$ 15.5 million in the last nine years in conservation programs across the planet. Brazilian projects received US$ 750,000 from that amount (3 %), distributed across 79 projects encompassing mammals (53 %), birds (18 %), reptiles (12 %), plants (7 %), amphibians (5 %), fishes (4 %), invertebrates (1 %), and fungi (0,004 %). The Fund uses the IUCN Red List as the primary guide to the conservation status of a given species. Taken altogether, these two funding opportunities directed more than US$ 25 million in the last decade specifically to fund the conservation of red listed species assessed in the IUCN. Being one of the most diverse countries in the world, and facing the biggest cuts ever on national science funding (Gibney 2015), why are Brazilian myriapod species, and probably many others, not eligible for international conservation grants?

Despite the IUCN being listed among the supporters of the 2014 Executive Summary of Brazil Red Book, the species listed in Brazil were not submitted to the IUCN global database. The Brazilian government invests in the elaboration of the national lists based on IUCN method and categories, but not taking this final step of submitting its assessments to the IUCN prevents international funding from being directed to Brazilian species. If a given species is classified as threatened in Brazil, but it is not listed in the IUCN Red List, it is not eligible for considerable international funding. Losing these important opportunities is a huge loss for Brazilian biodiversity conservation and for science, especially when investments are so scarce.

Fine scale red lists (i.e., country and state) are mandatory to know biodiversity and to plan short and mid-term conservation actions. However, consolidating those smaller pictures in a global database is also essential, because of their intrinsic value to science. For example, all the Myriapoda species assessed for the IUCN Red List are from Africa (98 %) and Southeast Asia (1 %). But myriapods are globally distributed, which suggests that there is a huge geographic gap in Myriapoda assessed data in the IUCN. As the endemic Brazilian myriapods were already assessed according to IUCN criteria but the data have not been yet sent to IUCN, analyzing the IUCN Red List alone could led to an erroneous conclusion that myriapods are only threatened in Africa and Southeast Asia. Besides that, Brazilian data have a significant impact on the knowledge of threatened Myriapoda considering also the diversity of the group, as the Brazilian Red List encompasses more families and genera than the IUCN Red List, especially for Diplopoda species. Then, adding national data to IUCN global database increases scientific knowledge of a given group, as it gathers scattered information into a single source. Second (and in a more applied sense), consolidating those smaller pictures in a global database is important to concentrate efforts for biodiversity conservation allowing priorities to be set at a global scale – which, in the red list case, would include countries’ red lists information which is not yet encompassed by IUCN global database. Besides that, it also allows endemic threatened species to be eligible for international funding. Then, countries that elaborate their national red lists based on IUCN methods (guidelines are available at its website) must take the final step of submitting their data to the IUCN staff for validation and inclusion in the red list. Submitting national red lists data to IUCN allows countries to achieve international funding and also helps to fill in the gaps in biodiversity knowledge and in the IUCN global database.

### Myriapoda threatened species in Brazilian protected areas

The myriapod species in the Brazilian Red List are not widely distributed across Brazil. Our results show that there are more threatened species in threatened habitats. Among threatened myriapods, 40 % are in the Atlantic Forest, and 33 % in the Cerrado – the biomes with the lowest proportion of remaining vegetation in Brazil: 8.5 % (MMA 2017) and 45 % (Coura et al. 2011), respectively. Oliveira et al. (2017) found that most species of vertebrates, arthropods (including millipedes) and angiosperms in their dataset had less than 30 % of their geographical distribution within Brazilian PAs. Our results, which include centipedes, and exclude non-myriapod groups, are consistent with theirs, as only 33 % of species among the threatened Brazilian Myriapoda occur inside PAs. Added to these low percentages there are PADDD events (downgrading, degazettement or downsizing of PAs) and intense and unregulated tourism representing great threats to biodiversity conservation within PAs in Brazil. In fact, there is an urgent call to designate new PAs in the Atlantic Forest and the Cerrado to prevent species loss due to the potential impact of the human population growth and agricultural expansion (Junk et al. 2006, Overbeck et al. 2015). The Cerrado, particularly, is the most coveted biome for agribusiness expansion (Overbeck et al. 2015, Strassburg et al. 2017). Even though invertebrates play essential ecological roles in ecosystem functioning, the pollination function developed by bees is probably the most common argument for conserving invertebrates. In Brazil, there are two cases of PAs created for invertebrate’s conservation (the velvet worm *Epiperipatus acacioi* (Marcus and Marcus 1955) and dragonfly communities), both PAs in Brazil Southeast. However, these are clearly exceptions in the Brazilian conservation agenda. Unfortunately, without the creation of PAs and protection of the threatened myriapod species, their extinction becomes more probable.

Additionally, the majority of threatened Myriapoda species is only known for subterranean habitats, considered as fragile environments with a high degree of endemism and morphological, ecological, and behavioral specialization among its communities (Bichuette and Trajano 2010). Among many aspects of nature that have a great potential for tourism, caves stand out due to their unique features, both scientific and esthetic, resulting in a high degree of attractiveness (Lobo et al. 2013). However, excessive human visitation is pointed as one of the major causes of impact for subterranean faunas, as a result of the considerable development of speleology as sport and adventure, overcrowding many caves (Bichuette and Trajano 2010). Being at the same time fragile and attractive, cave conservation turns to be a huge challenge concerning whole endemic invertebrate communities. The IUCN (1992) lists tourism as the second major threat to protected areas (after exotic fauna). Globally, terrestrial PAs receive approximately 8 billion visits per year (Balmford et al. 2015). On the other hand, Brazil’s national parks received 6.5 million visits in 2014 (Castro et al. 2015). Tourism related to nature or wildlife is a rapidly growing economic activity, especially in developing countries, which are more biodiverse and where it can generate income for local communities and governments (Curtin and Kragh 2014). Then, the lack of management plans in PAs represents a barrier to the development of ecotourism (Tortato and Izzo 2017). If carefully planned, managed and controlled, ecotourism in caves can minimize or even avoid most negative effects (Gossling 1999), and generate economic opportunities for local communities. For example, the economic benefits accrued from jaguar observation tourism far outweighed the costs of cattle losses in private ranches in Brazil, where local people still engage in the persecution and killing of large cats (Tortato et al. 2017). So, even if controversial, cave ecotourism can contribute to safeguard biodiversity and ecosystem functions in developing countries, even though meeting the requirements for ecotourism is extremely difficult (Gossling 1999).

The whole picture of PAs in Brazil, considering both the PADDD events and unregulated tourism, suggests that the PAs network in Brazil tends to fail in conserving biodiversity and needs to be strengthened to achieve conservation goals in the long run. However, the political scenario in Brazil is not optimistic. Ironically, politicians defending the agriculture industry, hydropower system and mineral extraction expansion have a strong influence on environmental political decisions in Brazil, and frequently succeed in getting polemic decisions quickly approved without public and technical consultations (Fearnside 2015). It seems that mineral extraction pressure will not cool down in the near future in Brazil, considering national government’s recent proposition of attracting private investments to explore minerals in the Amazon, among other measures of the Brazilian Mineral Industry Revitalization Program (DNPM 2017). Then, our analysis suggests that Myriapoda species extinction risks are likely to be worse than those stated in the 2014 Brazilian Red List, once the high number of Data Deficient species (42 %) may hide a significant number of species in threatened conditions. Besides, this scene may be similar, or worse, when considering other invertebrate groups. The total number of myriapods assessed for the 2014 Brazilian Red List represents 35% of all species registered from Brazil of its two major classes (Chilopoda and Diplopoda). The proportion of other invertebrate groups assessed was much smaller, such as Lepidoptera (3 %), Hymenoptera (3 %), Arachnida (2 %), and Coleoptera (0.005%). These important data gaps in scientific knowledge probably hide a significant number of terrestrial invertebrate species not being protected by the Brazilian PAs system. The current PA system fails to protect the majority of endemic species in Brazil (Oliveira et al. 2017) and here it also fails when considering Myriapoda endemic species in Brazil, and likely other terrestrial invertebrate groups.

Then, rather than a specific case for Myriapoda and for Brazil, the points discussed here can be related to arthropods (Lewinsohn et al. 2005, Diniz-Filho et al. 2010, Cardoso et al. 2011) and for the tropics, as most diverse countries are mainly emerging economies facing similar challenges in assessing species extinction risks, PAs network management, and science funding. Therefore, we recommend:

– Investing in taxonomic and ecological studies concerning myriapods and other arthropods in the tropics;

– Investing in biodiversity inventories within PAs networks in the tropics;

– Stimulating individual countries to submit their national red lists data to the IUCN.

## Supplementary materials

Supplementary material 1
Myriapoda threatened species geographic coordinates Data type: occurence This is an open access article distributed under the terms of the Creative Commons Attribution License (CC BY 4.0), which permits unrestricted use, distribution, and reproduction in any medium, provided the original author and source are credited.Manoela Karam-Gemael, Thiago Junqueira Izzo, Amazonas Chagas-Jr
